# Blood Volume, Hemoglobin Mass, and Peak Oxygen Uptake in Older Adults: The Generation 100 Study

**DOI:** 10.3389/fspor.2021.638139

**Published:** 2021-04-01

**Authors:** Kari Margrethe Lundgren, Nils Petter Aspvik, Knut Asbjørn Rise Langlo, Tonje Braaten, Ulrik Wisløff, Dorthe Stensvold, Trine Karlsen

**Affiliations:** ^1^Cardiac Exercise Research Group, Department of Circulation and Medical Imaging, Norwegian University of Science and Technology (NTNU), Trondheim, Norway; ^2^Department of Sociology and Political Science, Norwegian University of Science and Technology (NTNU), Trondheim, Norway; ^3^Department of Community Medicine, University of Tromsø - The Arctic University of Norway, Tromsø, Norway; ^4^Faculty of Nursing and Health Sciences, Nord University, Bodø, Norway; ^5^School of Human Movement and Nutrition Science, University of Queensland, Brisbane, QLD, Australia

**Keywords:** VO_2_ peak, plasma volume, Hb-mass, erythrocyte volume, aging, Gen 100

## Abstract

**Purpose:** To investigate the association between blood volume, hemoglobin mass (Hb_mass_), and peak oxygen uptake (VO_2peak_) in healthy older adults.

**Methods:** Fifty fit or unfit participants from the prospective randomized Generation 100 Study (*n* = 1,566) were included (age- and sex-specific VO_2peak_ above or below average values). Blood, plasma, and erythrocyte volume and Hb_mass_ were tested using the carbon monoxide rebreathing method within 1 week after VO_2peak_ testing.

**Results:** Mean age, BMI, Hb_mass_, blood volume, and VO_2peak_ were 73.0 ± 2.1 years, 24.8 ± 3.3 kg·m^2^, 10.0 ± 1.7 g·kg^−1^, 76.4 ± 11.8 mL·kg^−1^, and 33.5 ± 8.4 mL·kg^−1^·min^−1^. VO_2peak_ in fit and unfit participants and women and men were 38.6 ± 6.5 and 25.8 ± 3.8 mL·kg^−1^·min^−1^, 30.7 ± 7.6 mL·kg^−1^·min^−1^, and 35.5 ± 8.5 mL·kg^−1^·min^−1^, respectively. Women were shorter (Δ14 cm), leaner (Δ13 kg), and with less muscle mass (Δ9%) than men (*P* < 0.05). Relative erythrocyte volume and Hb_mass_ were lower in women, and blood and erythrocyte volume and Hb_mass_ were higher in the fit participants (*P* < 0.05). Hb_mass_ and erythrocyte volume explained 40 and 37%, respectively, of the variability in VO_2peak_, with a limited effect of physical-activity adjustment (40 and 38%, respectively). Blood and plasma volume explained 15 and 25%, respectively, of VO_2peak_ variability, and the association was strengthened adjusting for physical activity (25 and 31%, respectively), indicating a training-dependent adaptation in plasma but not erythrocyte volume (*p* ≤ 0.006).

**Conclusions:** Blood and plasma volumes were moderately associated with VO_2peak_ in healthy older men and women, and the association was strengthened after adjustment for physical activity. Hb_mass_ and erythrocyte volume were strongly associated with VO_2peak_ but unrelated to physical activity.

## Introduction

Both blood volume and hemoglobin mass are positively associated with endurance performance and maximal oxygen uptake (VO_2max_) (Davy and Seals, 1994; Stevenson et al., 1994; Heinicke et al., 2001). This association has been investigated extensively in elite endurance athletes but to a lesser extent in healthy older people with age-dependent physical activity and fitness levels (Aspenes et al., 2011; Hansen et al., 2019).

Elite endurance athletes have higher blood volume compared to less well-trained endurance athletes, strength and power athletes, and recreational athletes with lower fitness levels (Heinicke et al., 2001; Schmidt and Prommer, 2008), and blood volume and total hemoglobin mass explain about 60 and 50% of variability in VO_2max_, respectively (Heinicke et al., 2001). Elite endurance athletes, matched for training and endurance performance levels, have similar levels of blood volume and hemoglobin mass, despite large differences in exercise-training modes (kayaking, running, or cross-country skiing) (Lundgren et al., 2015). A high VO_2max_ without any training history has been found to be due to a high and hemodynamically active blood volume in young men (Martino et al., 2002). In people with spinal-cord injuries, elevated hemoglobin mass is seen in athletes compared to inactive people (Schumacher et al., 2009), and exercise training increased both total blood volume and hemoglobin mass in previously untrained spinal-cord-injured people (Houtman et al., 2000). In addition, both detraining and bed rest are associated with lower blood volume and red cell mass and linked to reduced heart function and VO_2max_ (Coyle et al., 1986; Convertino, 1997).

It is well-known that VO_2max_ decreases with age (~6–9%·decade^−1^) and is exercise training dependent (Andersen and Hermansen, 1965; Hermansen, 1973; Stevenson et al., 1994; Aspenes et al., 2011; Edvardsen et al., 2013). In comparison to younger people, older men and women have reduced blood and erythrocyte volumes (14–24%) (Davy and Seals, 1994; Stevenson et al., 1994). On the other hand, in a case report of an 80-year-old man with a high physical-activity level and a world-record VO_2max_ for his age, his blood volume and hemoglobin mass (Karlsen et al., 2015) correspond to the levels of recreational athletes in their twenties (Schmidt and Prommer, 2008). Still, it remains unclear whether the levels of blood volume and hemoglobin mass are the results of physiological adaptation to sea-level exercise or a distinct phenotype predisposed for successful endurance athletic performance (Heinicke et al., 2001).

To our knowledge, no study has previously investigated the association between blood volume and hemoglobin mass with VO_2peak_ in people aged 70 years and older. Therefore, the primary aim of this study was to investigate the association between blood volume and VO_2peak_ in men and women 70–77 years of age. Secondary aims were to investigate the association between hemoglobin mass and erythrocyte volume with VO_2peak_. We hypothesized blood volume to be positively associated with VO_2peak_ in 70–77-year-old people.

## Materials and Methods

### Participants

A total of 53 participants in the Generation 100 Study (ntnu.edu/cerg/generation100) (Stensvold et al., 2015) volunteered for this cross-sectional substudy and had their blood volume and total hemoglobin mass measured within 1 week of the VO_2peak_ test. Based on VO_2peak_ performance, participants were selected based on being unfit with a VO_2peak_ below 24 ml·kg^−1^·min^−1^ for women and 31 ml·kg^−1^·min^−1^ for men and being fit with a VO_2peak_ above 29 ml·kg^−1^·min^−1^ and 38 ml·kg^−1^·min^−1^ for women and men, respectively. The selection cutoffs were determined based on mean age- and sex-specific values from the HUNT Fitness Study (Aspenes et al., 2011). The selection was made to secure study VO_2peak_ diversity. Inclusion criteria in addition to VO_2peak_ were the ability to undergo the blood-volume examination within a week after a VO_2peak_ test and, otherwise, being in good health. Exclusion criteria for participation were self-reported significant blood loss (≥500 mL) within the last 3 months, anemia (according to the methodology cutoff recommendations of ≤ 13 g·dL^−1^ for men and ≤ 11 g·dL^−1^ for women), 10 or more days of altitude training or altitude living during the past 3 months, any self-reported chronic kidney disease, cardiovascular or pulmonary disease, or medication limiting maximal endurance performance. The investigation was conducted from 2012 to 2014. Three participants were excluded from the data analysis due to missing data.

### Ethical Statement

The Generation 100 Study and the current substudy are approved by the Regional Committee for Medical Research Ethics, Norway (#2012/381-3 and #2012/1243, respectively) and are registered in the Clinical Trial Database (NCTO#1666340). All participants received oral and written information about the studies before signing the informed-consent forms. The studies were conducted in conformity with the policy statement and use of human participants of the Declaration of Helsinki.

### Study Design

The Generation 100 Study is a single-center, randomized, controlled phase IIb clinical trial designed to evaluate the effect of 5 years of exercise training on mortality in a population of older adults. The full study protocol (Stensvold et al., 2015) and a detailed description of cardiopulmonary exercise testing and report of reference data for VO_2peak_ and the primary study outcome in the cohort have been published (Stensvold et al., 2017, 2020). Briefly, the Generation 100 Study invited all men and women residents of Trondheim, Norway, born between 1936 and 1942 to participate (*n* = 6,966); a total of 1,567 participants were included between August 2012 and June 2013 and randomized 1:1 to either 5 years in an exercise-training group or to a control group encouraged to follow national recommendations for physical activity.

In this cross-sectional study, based on the initial VO_2peak_ results, eligible participants were asked to participate in the substudy. Within 1 week of the VO_2peak_ test, blood volume and hemoglobin mass were measured using a CO-rebreathing method (Schmidt and Prommer, 2005).

### Anthropometric Measurements

Body mass and body composition were assessed once in each participant by bioelectrical impedance analysis to the nearest 0.1 kg (InBody 720, Biospace CO, Ltd, Seoul, Korea). Height was measured to the nearest millimeter using a stadiometer (Seca 222, Hamburg, Germany). Resting blood pressure was measured in the fasting state with participants sitting calmly in quiet conditions. Three measurements were performed at 1-min intervals and blood pressure defined as the average of the last two measurements (Philips IntelliVue MP50, Philips Medizin Systeme, Boeblingen, Germany); blood samples were obtained from an arm vein. Serum triglycerides; total, LDL, and HDL cholesterol; high-sensitivity C-reactive protein (hsCRP); and glycolated hemoglobin (HbA1c) were measured using routine hospital protocols at the Department of Medical Biochemistry, St. Olav's University Hospital, Trondheim, Norway (Table 1).

**Table 1:** Participant characteristics.

	**Total group** **(*n* = 50)**	**Women** **(*n* = 21)**	**Men** **(*n* = 29)**
Age (years)	73.0, 2.1	72.7, 2.2	73.2, 2.1
Height (cm)	171.0, 9.0	162.7, 4.7^*^	176.9, 6.3
Weight (kg)	72.8, 12.4	65.3, 10.6^*^	78.2, 10.8
Fat-free mass (kg)	52.7, 9.5	43.6, 3.8^*^	59.3, 6.3
BMI (kg·m^2^)	24.8, 3.3	24.7, 4.0	25.0, 2.9
Waist circumference (cm)	91.8, 11.1	87.7, 12.3^*^	94.8, 9.2
Fat mass (%)	27.3, 8.1	32.2, 8.5^*^	23.7, 5.5
Muscle mass (%)	28.9, 5.6	23.5, 2.2^*^	32.8, 3.6
SPB (mmHg)	134, 20	133, 21	135, 19
DBP (mmHg)	76, 10	72, 9	78, 10
Resting HR (beats·min^−1^)	63.0, 10.4	64.1, 9.4	62.2, 11.2
Glucose (mmol·L^−1^)	5.71, 0.88	5.68, 0.70	5.73, 1.01
HbA1c (%)	5.66, 0.47	6.00, 0.37	5.70, 0.53
Total cholesterol (mmol·L^−1^)	5.83, 1.02	6.06, 0.90	5.66, 1.09
LDL (mmol·L^−1^)	3.62, 1.02	3.61, 0.92	3.63, 1.11
HDL (mmol·L^−1^)	1.71, 0.51	2.01, 0.56^*^	1.49, 0.35
Triglyceride (mmol·L^−1^)	1.10, 0.49	0.97, 0.34	1.19, 0.57
hs-CRP (mg·L^−1^)	1.93, 2.36	1.75, 2.20	2.06, 2.51
Hb (g·dl^−1^)	14.4, 1.1	13.7, 0.6^*^	14.9, 1.2
HCT (%)	42.5, 3.0	40.7, 1.7^*^	43.9, 3.0
Uniaxial CPM	271, 153	264, 118	275, 177
Triaxial CPM	508, 203	515, 178	502, 223
Steps	7, 082, 3, 483	6, 903, 3, 475	7, 209, 3, 558
PA-index 20 years-old	6.20, 3.32	5.90, 3.59	6.41, 3.16
PA-index 40 years old	5.80, 3.03	5.05, 3.34	6.34, 2.72
**Weekly physical activity frequency, 20 years old**			
Never (%)	2.0	0	3.4
Less than once a week (%)	6.0	9.5	3.4
Once a week (%)	28.0	33.3	24.1
2–3 times week (%)	32.0	23.8	37.9
Nearly every day (%)	32.0	33.3	31.0
**Weekly physical activity frequency, 40 years old**			
Never (%)	0.0	0.0	0.0
Less than once a week (%)	10.0	14.3	6.9
Once a week (%)	32.0	38.1	27.6
2–3 times week (%)	36.0	33.3	37.9
Nearly every day (%)	22.0	14.3	27.6

Data are mean ± standard deviation.

* *Between group p ≤ 0.05, CPM, counts·min^−1^; SPB, systolic blood pressure; DBP, diastolic blood pressure; HbA1c, glycolated hemoglobin, type A1c; LDL, low-density lipoprotein; HDL, high-density lipoprotein; hs-CRP, high-sensitivity C-reactive protein; Hb, hemoglobin; HCT, hematocrit; PA-index = physical activity index*.

### Blood-Volume Measurements

Blood volume, plasma volume, erythrocyte volume, and hemoglobin mass (Hb_mass_) were determined by a CO-rebreathing method (Bayreuth, Germany) (Schmidt and Prommer, 2005; Prommer and Schmidt, 2007). Participants were tested in the morning (between 08:00 and 10:00 a.m.) after an overnight fast (≥12 h) and were asked to drink 500 mL of water 2 h prior to the investigation. After 15 min of seated rest and a thorough explanation of the test procedures, two 30-μl capillary blood samples were collected from the fingertip before the participants exhaled air from the lungs followed by an inhalation of one bolus of 99.9% carbon monoxide gas from a spirometer setup. The bolus was 45–65 mL and estimated based on body mass and training state. The volume of carbon dioxide gas was estimated according to the measurement protocol with a factor of 0.7 and 0.8 kg^−1^ of their body weight for untrained women and men, respectively (Schmidt and Prommer, 2005). After inhaling the bolus of CO gas (99.9%), participants held their breath for 10 s and continued to rebreathe the gas mixture together with 3 L of 100% medical-grade oxygen gas in the spirometer for 2 min. After 2 min of rebreathing, they performed a full exhalation into the spirometer, and the spirometer was sealed for later analysis of gas content and volume (Draeger®, Luebeck, Germany). 30-μl capillary blood samples were collected from the fingertip again at 6 and 8 min after the start of rebreathing.

Capillary blood was analyzed immediately for HbCO% using the ABL800 FLEX analyzer (Radiometer Medical ApS, Brønshøj, Denmark). A 3-mL EDTA tube was collected from the antecubital fossa vein before rebreathing for hematocrit and hemoglobin analysis according to standard procedures at the Department of Medical Biochemistry, St. Olav's University Hospital, Trondheim, Norway (Sysmex XE-2100 analyzer, Sysmex Co., Kobe, Japan).

End-tidal CO concentration was measured with a CO gas tester (Draeger®, Luebeck, Germany) prior to and 4 min after the CO inhalation when participants were asked to perform a full expiration into a mouthpiece connected to the CO-gas tester. Blood volume, Hb_mass_, erythrocyte volume, and plasma volume were calculated using Spico Calculation Software 2.0 (Blood Tec, GbR, Bayreuth, Germany). A detailed description of the method can be found in the papers by Schmidt and Prommer (2005) and Prommer and Schmidt (2007). The participants performed and tolerated the procedure well. The typical error of the measurement in our laboratory was ~1.6% (unpublished data).

### Peak Oxygen Uptake

VO_2peak_ testing was performed at the core facility NeXt Move (ntnu.edu/mh/nextmove) at the Norwegian University of Science and Technology (NTNU), Trondheim. After an initial low-intensity warm-up period of 8–10 min, VO_2peak_ was measured in an incremental test to exhaustion using an indirect breath-by-breath ergospirometry system (METAMAX II, Leipzig, Germany). Data were analyzed using MetaSoft 3.9 (CORTEX Biophysik GmbH, Leipzig, Germany). The test was performed on a treadmill (PPS 55 Med, Woodway USA Inc, Waukesha, WI, USA) as a walking or running test depending on the participant's fitness level. After a brief treadmill customization phase and a 10-min warm-up, the first stage of the test was initiated at a workload derived from the warm-up treadmill incline and speed. The first stage was a 3-min steady-state phase, followed by a 2% increase in inclination for 2 min. After this, workload was increased by 1 km·h^−1^ or 2% inclination approximately every one and a half minute until exhaustion, and the mean of the three-highest consecutive 10 s measurements was used to determine VO_2peak_ (Stensvold et al., 2017). The criteria for achieving VO_2max_ was reached in 69% of the participants (plateau in oxygen uptake and a respiratory exchange ratio ≥1.05 at exhaustion) (Howley et al., 1995). As one-thirds of the participants in the study did not fulfill the predefined criteria for VO_2max_, we use the term VO_2peak_ over VO_2max_ in the study. Immediately after the test, the participants reported the rate of perceived exertion (RPE) according to the Borg scale in a range from 6 to 20 (Borg, 1973). Peak heart rate (HR_peak_) was measured by a heart-rate monitor (Polar RS400, Polar Electro Oy, Kempele, Finland), and HR_peak_ was determined by adding 5 beats·min^−1^ to the highest recorded value during the test (Berglund et al., 2019; Stensvold et al., 2020). Peak oxygen pulse (mL·beat^−1^) was calculated as VO_2peak_ (mL·min^−1^) divided by HR_peak_ (Wasserman, 2012). Heart-rate recovery was the change in heart rate from peak heart rate to the heart rate measured 1 min after stopping the test.

### Physical Activity

Objectively measured physical activity was assessed by the ActiGraph triaxial accelerometer GT3X+ (Manufacturing Technology, Inc, Florida, USA). After having completed clinical testing, participants were instructed to wear the monitor continuously for 7 days except when in contact with water. Data were accepted if the participants had a minimum of 4 days with recordings of at least 600 min per day. A detailed description of the physical-activity measurements and analysis is found elsewhere (Hall et al., 2014; Viken et al., 2016). Both uniaxial (vertical axis) and triaxial (vector magnitude) counts·min^−1^ (CPM) are reported in the current study (Santos-Lozano et al., 2013; Sun et al., 2013). Triaxial CPM is the most complete measure of physical activity with this methodology and was, therefore, chosen as the primary variable for physical-activity adjustments in association analysis of VO_2peak_ and blood-volume data. Steps (mean steps per day) were also assessed by the accelerometer. Physical activity at 20 and 40 years of age was retrospectively reported in a questionnaire at baseline. Participants were asked about physical-activity intensity and frequency at 20 and 40 years of age (Viken et al., 2016) and data used to generate a physical activity index for both 20 and 40 years of age. The following questions and response options with index scores were indexed: (1) How frequently did you exercise when you were 20 and 40 years old? Response options: never = 0, less than once a week = 0, once a week = 1, 2–3 times per week = 2, almost every day = 3. (2) If you were physically active once per week when you were 20 and 40 years old, how hard did you exercise (intensity of physical activity)? Response options: take it easy = 0, heavy breath and sweat = 5, push near exhaustion = 10 (Nes et al., 2011).

### Statistical Analysis

The participants' characteristics are presented as means and standard deviations by gender. Data normality was assessed using the Q-Q plot; all variables were found to be sufficiently normally distributed. For between-group comparisons, independent-sample *t*-tests were used. The association between blood-volume values and VO_2peak_ was tested using linear regression. The confounding effect of current physical activity (triaxial CPM), physical activity previous in life, and gender was controlled for by multiple regression analysis. The two-sided level of significance was set to *p* < 0.05. The associations were investigated in relative values (adjusted for body weight) as this controls for initial differences in body weight. All statistical analyses were performed using IBM SPSS Statistics software program version 26 (SPSS Inc. Chicago, IL, USA).

## Results

Relative Hb_mass_, erythrocyte, plasma, and blood volumes were positively associated with relative VO_2peak_ (Table 2). Hb_mass_ and erythrocyte volume accounted for ~40 and ~37% of the variability in VO_2peak_, respectively, while blood volume and plasma volume explained ~25 and ~15% of the variability in VO_2peak_, respectively. When adjusting for the effect of physical activity (triaxial counts per minute), the associations between VO_2peak_ and blood and plasma volumes were 5.2 and 10.0% stronger, with minor changes in the association with Hb_mass_ and erythrocyte volume. Heterogeneity by gender was assessed by the Wald test and showed no difference.

**Table 2:** Association between VO_2peak_ and blood-volume parameters.

**VO_**2peak**_ (mL·kg^**−1**^·min^**−1**^)**	**Hb_**mass**_ (g·kg^**−1**^)**	**EV (mL·kg^**−1**^)**	**PV (mL·kg^**−1**^)**	**BV (mL·kg^**−1**^)**
Unadjusted	*r*^2^ = 0.40 (*p* < 0.001) VO_2peak_ = 2.089+3.139β_1_	*r*^2^ = 0.37 (*p* < 0.001)VO_2peak_ = 4.156+0.990β_1_	*r*^2^ = 0.15 (*p* ≤ 0.006) VO_2peak_ = 13.121+0.435β_1_	*r*^2^ = 0.25 (*p* < 0.001)VO_2peak_ = 6.141+0.358β_1_
Adjusted for physical-activity	*r*^2^ = 0.40 (*p* < 0.001) VO_2peak_ = 2.098+ 2.548β_1_+0.012β_2_	*r*^2^ = 0.38 (*p* < 0.001)VO_2peak_ = 4.004+0.785β_1_+0.013β_2_	*r*^2^ = 0.25 (*p* < 0.005) VO_2peak_ = 11.636+0.313β_1_+0.015β_2_	*r*^2^ = 0.31 (*p* ≤ 0.001)VO_2peak_ = 6.430+0.267β_1_+0.014β_2_
Adjusted for physical activity and gender	*r*^2^ = 0.41 (*p* < 0.001) VO_2peak_ = 2.759+2.512β_1_+0.012β_2_ −0.242β_3_	*r*^2^ = 0.38 (*p* < 0.001)VO_2peak_ = 6.628+0.741β_1_+0.013β_2_-1.027β_3_	*r*^2^ = 0.25 (*p* ≤ 0.013) VO_2peak_ = 17.639+0.300β_1_+ 0.016β_2_-3.927β_3_	*r*^2^ = 0.31 (*p* ≤ 0.003)VO_2peak_ = 12.408+0.240β_1_+0.014β_2_-2.964β_3_
Adjusted for physical activity and PA-index 20-years old	*r*^2^ = 0.41 (*p* < 0.001) VO_2peak_ = 2.032+2.530β_1_+ 0.012β_2_+ 0.049β_4_	*r*^2^ = 0.38 (*p* < 0.001)VO_2peak_ = 3.855+0.776β_1_+ 0.012β_2_+0.083β_4_	*r*^2^ = 0.25 (*p* ≤ 0.012) VO_2peak_ = 10.776+0.308β_1_+0.015β_2_+ 0.228β_4_	*r*^2^ = 0.31 (*p* ≤ 0.003)VO_2peak_ = 5.954+0.262β_1_+ 0.013β_2_+0.173β_4_
Adjusted for physical activity and PA-index 40-years old	*r*^2^ = 0.49 (*p* < 0.001) VO_2peak_ = 3.039+1.943β_1_+ 0.013β_2_+ 0.795β_5_	*r*^2^ = 0.47 (*p* < 0.001)VO_2peak_ = 4.372+0.592β_1_+ 0.014β_2_+0.850β_5_	*r*^2^ = 0.42 (*p* < 0.001) VO_2peak_ = 7.643+0.262β_1_+ 0.016β_2_+ 1.088β_5_	*r*^2^ = 0.45 (*p* < 0.001)VO_2peak_ = 4.906+0.209β_1_+ 0.015β_2_+0.995β_5_

*Data are presented as adjusted and unadjusted r^2^ for the association between VO_2max_ and blood-volume parameters. VO_2peak_, peak oxygen uptake; Hb_mass_, hemoglobin mass; EV, erythrocyte volume; PV, plasma volume; BV, blood volume; PA, physical activity; β_1_, blood parameters; β_2_, physical activity; β_3_, gender; β_4_, retrospective physical activity, 20 years of age; β_5_, retrospective physical activity, 40 years of age*.

### Peak Oxygen Uptake

Women had lower peak VO_2_ (L·min^−1^ and mL·kg^−1^·min^−1^) and ventilation compared to men (*P* < 0.05) (Table 3). VO_2peak_ was 13.2 mL·kg^−1^·min^−1^ and 14.9 mL·kg_fatfree_^−1^ ·min^−1^ higher when comparing fit (39.0 ± 6.1 mL·kg^−1^·min^−1^, 52.0 ± 5.6 mL·kg_fatfree_^−1^ ·min^−1^) and unfit (25.8 ± 3.7 mL·kg^−1^·min^−1^, 37.1 ± 3.6 mL·kg_fatfree_^−1^ ·min^−1^) participants (*P* < 0.05), confirming VO_2peak_ inclusion diversity. The respiratory exchange ratio (RER) indicated equal test performance between men and women (Table 3) and fit (1.15 ± 0.06) and unfit participants (1.13 ± 0.10).

**Table 3:** Cardiopulmonary exercise test results.

	**Total group** **(*n* = 50)**	**Woman** **(*n* = 21)**	**Men** **(*n* = 29)**
VO_2peak_ (L·min^−1^)	2.41, 0.63	1.95, 0.32^*^	2.74, 0.59
VO_2peak_ (mL·kg^−1^·min^−1^)	33.5, 8.4	30.7, 7.6^*^	35.5, 8.5
VO_2peak_ (mL·kg_fatfree_^−1^ ·min^−1^)	45.8, 8.9	44.9, 7.3	46.4, 9.9
RER_peak_	1.15, 0.08	1.12, 0.07	1.16, 0.08
VE_peak_ (L·min^−1^)	90.4, 28.3	66.3, 12.9^*^	107.9, 23.0
HR_peak_ (beats·min^−1^)	163, 15	164, 14	162, 17
Heart-rate recovery (beats)	27.3, 11.6	25.9, 9.0	28.4, 13.2
SPB_peak_ (mmHg)	196, 28	201, 30	193, 29
DPB_peak_ (mmHg)	80, 13	77, 11	81, 15
BORG scale (6–20)	17.6, 1.5	17.5, 1.6	17.7, 1.4

Data are mean ± standard deviation.

* *Between group p ≤ 0.05. VO_2peak_, peak oxygen uptake; VE, ventilation; RER, respiratory exchange ratio; HR_peak_, peak heart rate; SPB_peak_, peak systolic blood pressure; DBP_peak_, peak diastolic blood pressure. Heart-rate recovery was the change in heart rate from peak heart rate to the heart rate measured 1 min after stopping the test*.

### Blood Volume and Total Hemoglobin Mass

Absolute blood, plasma, and erythrocyte volume, hemoglobin mass, relative hemoglobin mass (g·kg^−1^), and erythrocyte volume (mL·kg^−1^) were lower in women compared to in men. Plasma volume relative to fat-free mass was higher in women than in men (all *P* < 0.05) (Table 4). Absolute and relative body weight, blood, and erythrocyte volumes and total Hb_mass_ were higher in the fit (79.4 ± 11.2 mL·kg^−1^, 31.1 ± 4.6 mL·kg^−1^, 10.5 ± 1.5 g·kg^−1^) compared to the unfit 72.3 ± 11.6 mL·kg^−1^, 27.5 ± 5.2 mL·kg^−1^, 9.3 ± 1.8 g·kg^−1^) participants (all *P* < 0.05). Clinically measured hemoglobin and hematocrit were lower in women than in men (*P* < 0.05) (Table 1).

**Table 4:** Blood volume values.

	**Total group** **(*n* = 50)**	**Women** **(*n* = 21)**	**Men** **(*n* = 29)**
Hb_mass_ (g)	727, 161	583, 72^*^	832, 120
Blood volume (mL)	5532, 1028	4677, 662^*^	6152, 823
Plasma volume (mL)	3380, 586	2944, 357^*^	3695, 514
Erythrocyte volume (mL)	2152, 482	1733, 226^*^	2457, 376
Hb_mass_ (g·kg^−1^)	10.0, 1.7	9.1, 1.5^*^	10.7, 1.5
Erythrocyte volume (mL·kg^−1^)	29.6, 5.1	27.0, 4.8^*^	31.5, 4.6
Blood volume (mL·kg^−1^)	76.4, 11.8	73.0, 12.6	78.9, 10.7
Plasma volume (mL·kg^−1^)	46.8, 7.4	45.9, 8.0	47.4, 7.0
Hb_mass_ (g·kg_fatfree_^−1^)	13.8, 1.6	13.4, 1.6	14.0, 1.6
Erythrocyte volume (mL·kg_fatfree_^−1^)	40.8, 5.1	39.9, 5.1	41.5, 5.1
Blood volume (mL·kg_fatfree_^−1^)	105.6, 11.9	107.8, 13.2	103.9, 10.8
Plasma volume (mL·kg_fatfree_^−1^)	64.7, 8.1	67.9, 8.6^*^	62.4, 7.1

Data are mean ± standard deviation.

* *Between group p ≤ 0.05. VO_2max_, peak oxygen uptake; Hb_mass_, hemoglobin mass*.

### Physical Activity

There were no differences in current physical activity or retrospectively reported weekly physical activity index at 20 and 40 years of age between men and women (Table 1). There were no significant differences in the physical activity index at 20 and 40 years of age. Adjusting for the physical activity index at 20 years of age had no effects on the associations between the blood variables and VO_2peak_. Adjusting for physical activity index at 40 years of age gave a 8, 9, 17, and 14% stronger association between Hb_mass_, erythrocyte volume, plasma volume, and blood volume with VO_2peak_, respectively (Table 2). The fit participants were in 2012–2014 more physically active (Uniaxial CPM 314 ± 150, Triaxial CPM 571 ± 193, Steps 8,013 ± 3,212) than the unfit participants (Uniaxial CPM 202 ± 137, Triaxial CPM 408 ± 182, Steps 5,629 ± 3,486) (all *P* < 0.05). Self-reported physical activity indices at 20 and 40 years of age were equal between fit and unfit participants (*p* = 0.467).

### Participant Characteristics

Anthropometric characteristics and physical-activity levels are shown in Table 1. Women were shorter, leaner (in kg, kg_FFM_, waist circumference), with higher fat mass, and with less muscle mass than in men. Women had higher HDL, and lower hemoglobin and hematocrit than in men (*P* < 0.05 for all values). Adjusting for gender had no effects on the studied associations between relative VO_2peak_ and blood volume, plasma volume, erythrocyte volume, and Hb_mass_, respectively.

## Discussion

Our main finding is that both relative erythrocyte volume and Hb_mass_ were strongly associated with VO_2peak_ in healthy 70–77-year-olds in our sample, explaining ~40% of the variability in VO_2peak_. The association was unrelated to gender, objectively measured current physical activity measured in triaxial CPM, or self-reported physical activity at 20 years of age. Relative plasma and blood volumes were also associated with VO_2peak_, explaining ~15 and ~25% of the variability, respectively. The association increased by five percentage points for blood volume and 10 points for plasma volume when adjusted for participants' current level of physical activity, explaining ~30 and ~25% of the association with VO_2peak_, respectively. These associations were also unrelated to gender and physical activity at 20 years of age.

The association between VO_2peak_ and erythrocyte volume and Hb_mass_ is well-documented in several populations of athletes, untrained and trained, young and old people (Davy and Seals, 1994; Sawka et al., 2000; Heinicke et al., 2001; Koons et al., 2019). Blood volume and Hb_mass_ in our participants corresponded to what has previously been reported in elderly trained men, middle-aged to elderly trained and untrained women, and untrained young controls (Davy and Seals, 1994; Stevenson et al., 1994; Heinicke et al., 2001) but were lower than in younger-endurance athletes (Heinicke et al., 2001) and slightly higher than in middle-aged to older men and women (Koons et al., 2019). To the best of our knowledge, our study is the first to show that Hb_mass_ and erythrocyte volume are important determinants of VO_2peak_ also in 70–77-year-old people. In comparison to previous studies (Stevenson et al., 1994; Heinicke et al., 2001), Hb_mass_ and erythrocyte volume explained slightly less of the variability in VO_2peak_ in our study. The between-study differences could be due to genetic differences, different training levels, training modes, iron availability, and age as the association between Hb_mass_/erythrocyte volume and VO_2max_ varies between different athletic populations (Heinicke et al., 2001) and trained and untrained participants (Stevenson et al., 1994; Heinicke et al., 2001), as well as with age (Davy and Seals, 1994) and growth (Steiner et al., 2019; Landgraff and Hallen, 2020). The impact of Hb_mass_ and erythrocyte volume on VO_2peak_ was unrelated to the participants' current physical-activity levels and thereby more likely to be mainly genetically determined (Lundby and Robach, 2015; Montero and Lundby, 2017). This is in accordance with a no-exercise-dependent increase in red-cell mass with endurance-training interventions in people older than 60 years in a recent meta-analysis (Montero and Lundby, 2017), as well as no impact of exercise training on Hb_mass_ in longitudinal studies of children and adolescents (Steiner et al., 2019; Landgraff and Hallen, 2020).

VO_2max_ is strongly associated with exercise intensity (Rognmo et al., 2004; Helgerud et al., 2007), and in our study, both current physical activity (*r*^2^ = 0.17, *p* = 0.007) and the physical activity index at 40 years of age (*r*^2^ = 0.14, *p* = 0.007) were weakly associated with VO_2peak_. Adjusting for self-reported physical activity at 20 and 40 years of age thereby gave different effects on the VO_2peak_ associations. As previous studies have reported these blood variables to be unrelated to physical activity (Steiner et al., 2019; Landgraff and Hallen, 2020), it is challenging to explain why the physical activity index at 40 and 20 years of age affected the VO_2peak_ associations differently. The mean physical activity index was equal at 20 and 40 years of age (*p* = 0.467), and there were no differences between men and women. As physical activity was self-reported retrospective after over 30 and 50 years, we believe that the impact of historic physical activity in our study should be interpreted with caution.

In our male participants, Hb_mass_ relative to fat-free mass was equal to that reported in trained and untrained 19-year-old men (Steiner et al., 2019), while our women had slightly higher values than in 15-year-old girls (Landgraff and Hallen, 2020). As our data is comparable to previous studies in younger populations, and an increase in Hb_mass_ from 12 to 15 years of age was found to be associated with the increase in fat-free mass with growth (Landgraff and Hallen, 2020), it might indicate that a stable Hb_mass_ exists in relation to lean body mass throughout the lifespan (Steiner et al., 2019; Landgraff and Hallen, 2020). Fat-free mass in our study was slightly higher than in a large Danish cohort study that shows lean body mass to remain relatively unchanged until after 70 years of age in men and even later for women. This indicates that it is feasible to remain physically active and maintain physical function as a septuagenarian (Suetta et al., 2019). Relative to fat-free mass, we found no difference in Hb_mass_ between men and women. This is different from higher Hb_mass_ found in 15-year-old boys compared to that in girls (Landgraff and Hallen, 2020), but as Hb_mass_ was found to increase between 16 and 19 years of age in young men (Steiner et al., 2019), age could explain the between-study difference. As anemic participants were excluded, a normal erythropoiesis should be expected in all our participants (Shoemaker et al., 1996); thus, any interaction between erythropoiesis and other hormones known to fluctuate with physical activity or to be affected by aging (e.g., reduced testosterone) (Feldman et al., 2002) remains unaccounted for in this study (Montero and Lundby, 2018). As all the studies have a moderate number of participants, differences between studies could also be due to individual variability.

It is well-established that regular physical activity causes training-induced hypervolemia (Montero and Lundby, 2017; Steiner et al., 2019; Landgraff and Hallen, 2020) with positive effects on thermoregulation, heart rate, and stroke volume and may also have a cardioprotective effect (Warburton et al., 2004; Convertino, 2007). Compared to a study of middle-aged to older men and women, both blood volume and VO_2peak_ relative to fat-free mass were higher in our participants (Carrick-Ranson et al., 2013). As current physical activity enhanced the association between VO_2peak_ and plasma volume by ~10 percentage points, our data might suggest that the plasma-volume component of blood volume is influenced by regular physical activity even in 70–77-year-old people (Montero and Lundby, 2017, 2018; Steiner et al., 2019). In a study of the association between blood volume and VO_2max_ with aging, physical activity tended to attenuate an expected decrease in blood volume and VO_2max_ with aging in women (Koons et al., 2019), supporting our finding of the importance of physical activity for maintaining both blood volume and VO_2max_ with increasing age. Adjusting for self-reported physical activity at 20 and 40 years of age affected the associations between VO_2peak_ and blood and plasma volumes differently. As with Hb_mass_, it is challenging to explain this discrepancy, but as physical activity was self-reported retrospective after over 30 and 50 years, we believe the data should be interpreted with caution.

VO_2max_ is an important prognostic factor for cardiovascular disease and mortality (Myers et al., 2002, 2015; Kodama et al., 2009). Several factors are known to limit VO_2max_, including muscular, vascular, and cardiac function (Convertino, 1991; Richardson, 2000; Wagner, 2000; Heinicke et al., 2001; Schmidt and Prommer, 2008). Muscle strength and vascular and cardiac function were not measured in our study; however, our results indicate that hemoglobin-dependent oxygen transport is the strongest determinant of VO_2peak_ measured in the present study (Sawka et al., 2000; Montero and Lundby, 2017), most likely due to genetic predispositions since controlling for objectively measured current physical activity did not alter the association with VO_2peak_ (Montero and Lundby, 2017). The effect of exercise-induced hypervolemia has been shown to increase the heart's diastolic function, explaining elements of exercise-induced increases in VO_2max_ (Warburton et al., 2004) and adding data to the importance of regular physical activity for maintaining a healthy heart and cardiovascular function in aging. Several factors regulate vascular volume, including exercise, electrolyte concentration, blood-protein content, diuretic hormones, and age- or exercise-associated kidney function (Convertino, 2007; Robinson-Cohen et al., 2009; Montero and Lundby, 2018). Despite higher-indexed blood volumes, the fit participants in our study did not display higher blood pressure than the unfit participants. The mechanism by which the fit participants maintained normal blood pressure despite higher blood volumes might be through enhanced endothelial function and lower vascular tone, allowing for better organ perfusion and cardiovascular health (Boreham et al., 2004; Seals et al., 2008). Our participants were selected based on their VO_2max_ test results, in addition to their being healthy with normal self-reported kidney function and no anemia. Creatinine or other measures of kidney function were not tested; thereby, we cannot rule out that deteriorating kidney function with older age, physical activity, or fitness level might have affected vascular volume among our participants. However, the likelihood is minor due to both a thorough health screening at inclusion in the Generation 100 Study, normal blood pressure, and good self-reported health status including kidney function.

Several studies have suggested that total blood volume plays a minor role in the age-related decline in VO_2max_ (Koons et al., 2019) and that the relationship is constant in relation to metabolically active muscle tissue (Carrick-Ranson et al., 2013). Muscle strength and muscle mass are known to decrease with increasing age (Karlsen et al., 2017); this might explain the discrepancy between studies reporting a strong correlation between blood volume and VO_2max_ and no age-related decline in blood volume (Jones et al., 1997) or reduction in blood volume, plasma volume, and red-cell volume as seen in older men (Davy and Seals, 1994). There were no differences in muscle mass in fit and unfit participants in our study; therefore, blood volume is most likely unassociated with skeletal muscle mass. VO_2peak_ in our 70–77-year-old participants is, thus, most likely explained by genetically determined Hb_mass_/erythrocyte volume, current physical-activity-associated plasma volume levels, or possibly other mechanisms beyond our control in this study.

### Strengths and Limitations

A strength of this study is its inclusion and testing of both men and women older than 70 years of age, as this population group is often excluded from studies. Also, few studies to date have reported data on blood volume and Hb_mass_ in women (Montero and Lundby, 2017). With a thorough screening in the Generation 100 Study, we had good control over participants' demographics, including anemia; therefore, participants with below-normal red blood-cell levels did not confound the physiological measurements. The use of gold-standard VO_2peak_ testing as well as adjustments for objectively measured current physical activity strengthens the associations in the study. No significant group difference was found in peak RER, and both group means were above 1.1, indicating the same level of exhaustion during VO_2peak_ testing in the participants. The BORG scale was above 17 in both groups, indicating high test effort and exhaustion.

The study has several limitations, including the number of participants and gender variability. In addition, the selection of participants could have caused bias in the data material as additional testing could be considered more valuable to some but not all participants. It was easier to recruit participants with higher than lower VO_2peak_, resulting in fewer participants with low VO_2peak_, as well as fewer women. However, the objectively measured physical-activity data are comparable to the complete study group (Aspvik et al., 2016) and another comparable study (Lohne-Seiler et al., 2014), indicating minor selection bias in the substudy. The main reason for the VO_2peak_ difference between cohorts is most likely the different selection of participants between studies (Aspenes et al., 2011; Stensvold et al., 2017). Neither ferritin, iron, creatinine, testosterone, nor erythropoietin were measured in our study; hence, any genetic predisposition to blood-cell turnover, age-dependent reduction in testosterone, or reduced estimated glomular filtration rate (eGFR) in association with older age was not studied. In addition, data on other lifestyle factors throughout life, and objectively measured physical activity at 20 and 40 years of age would have benefited the study. The CO-rebreathing methodology has been used for over 100 years; still the methodology is under investigation and development (Keiser et al., 2013).

## Practical Implications

Our study indicates that blood and plasma volumes are affected by current physical activity in older adult lowlanders, with erythrocyte volume and hemoglobin mass possibly more genetically determined by other lifestyle factors earlier in life.

## Conclusion

Blood and plasma volumes were moderately associated with VO_2peak_ in lowland-dwelling healthy older men and women, and the association was strengthened after adjustment for physical activity. Hemoglobin mass and erythrocyte volume were strongly associated with VO_2peak_ but unrelated to current daily physical activity.

## Data Availability Statement

The datasets presented in this article are not readily available because not part of original Ethical approval. Requests to access the datasets should be directed to trine.karlsen@nord.no.

## Ethics Statement

The studies involving human participants were reviewed and approved by Regional Committee for Medical Research Ethics, Norway (#2012/381-3 and #2012/1243, respectively) and are registered in the Clinical Trial Database (NCTO#1666340). The patients/participants provided their written informed consent to participate in this study.

## Author Contributions

All authors assisted in the writing of the manuscript and were involved in the study design and/or data collection, analysis, and interpretation.

## Conflict of Interest

The authors declare that the research was conducted in the absence of any commercial or financial relationships that could be construed as a potential conflict of interest.
